# CyberKnife^®^ fixed cone and Iris™ defined small radiation fields: Assessment with a high‐resolution solid‐state detector array

**DOI:** 10.1002/acm2.12414

**Published:** 2018-07-12

**Authors:** Giordano Biasi, Marco Petasecca, Susanna Guatelli, Ebert A. Martin, Garry Grogan, Benjamin Hug, Jonathan Lane, Vladimir Perevertaylo, Tomas Kron, Anatoly B. Rosenfeld

**Affiliations:** ^1^ Centre for Medical Radiation Physics University of Wollongong Wollongong 2522 NSW Australia; ^2^ Department of Radiation Oncology Sir Charles Gairdner Hospital Nedlands WA Australia; ^3^ School of Physics and Astrophysics University of Western Australia Crawley WA Australia; ^4^ SPA‐BIT Kiev Ukraine; ^5^ Peter MacCallum Cancer Centre Melbourne VIC Australia; ^6^ Sir Peter MacCallum Cancer Institute University of Melbourne Melbourne VIC Australia

**Keywords:** CyberKnife, SRT, quality assurance, small‐field dosimetry, 2D monolithic silicon array detector

## Abstract

**Purpose:**

The challenges of accurate dosimetry for stereotactic radiotherapy (SRT) with small unflattened radiation fields have been widely reported in the literature. In this case, suitable dosimeters would have to offer a submillimeter spatial resolution. The CyberKnife^®^ (Accuray Inc., Sunnyvale, CA, USA) is an SRT‐dedicated linear accelerator (linac), which can deliver treatments with submillimeter positional accuracy using circular fields. Beams are delivered with the desired field size using fixed cones, the InCise™ multileaf collimator or a dynamic variable‐aperture Iris™ collimator. The latter, allowing for field sizes to be varied during treatment delivery, has the potential to decrease treatment time, but its reproducibility in terms of output factors (OFs) and dose profiles (DPs) needs to be verified.

**Methods:**

A 2D monolithic silicon array detector, the “Octa”, was evaluated for dosimetric quality assurance (QA) for a CyberKnife system. OFs, DPs, percentage depth‐dose (PDD) and tissue maximum ratio (TMR) were investigated, and results were benchmarked against the PTW SRS diode. Cross‐plane, in‐plane and 2 diagonal dose profiles were measured simultaneously with high spatial resolution (0.3 mm). Monte Carlo (MC) simulations with a GEANT4 (GEometry ANd Tracking 4) tool‐kit were added to the study to support the experimental characterization of the detector response.

**Results:**

For fixed cones and the Iris, for all field sizes investigated in the range between 5 and 60 mm diameter, OFs, PDDs, TMRs, and DPs in terms of FWHM measured by the Octa were accurate within 3% when benchmarked against the SRS diode and MC calculations.

**Conclusions:**

The Octa was shown to be an accurate dosimeter for measurements with a 6 MV FFF beam delivered with a CyberKnife system. The detector enabled real‐time dosimetric verification for the variable aperture Iris collimator, yielding OFs and DPs consistent with those obtained with alternative methods.

## 1 INTRODUCTION

**Figure 1:**
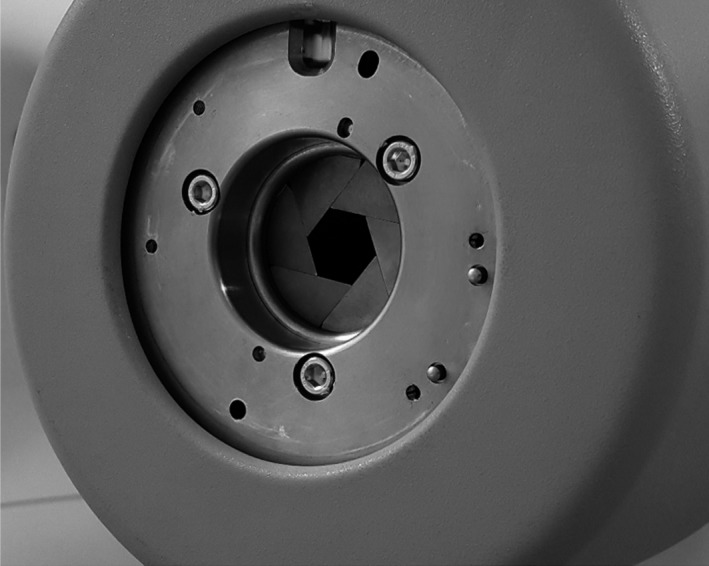
Snapshot of the CyberKnife linac head with a variable aperture Iris collimator at 40 mm diameter.

The CyberKnife^®^ system can deliver stereotactic radiotherapy (SRT) treatments with high doses in a few fractions using small radiation fields, with submillimeter positional accuracy.^1^, ^2^ The linear accelerator (linac), mounted on a robotic arm, is operated without a flattening filter and the treatment beam is shaped using fixed circular cones, the InCise™ multileaf collimator or the variable aperture Iris™ collimator (Fig. 1).^1^, ^3^ The latter, allowing for the radiation field size to be varied during treatment delivery, has the potential to decrease the peripheral dose compared to fixed collimators^4^ and to reduce treatment time.^3^ A CyberKnife system, the first of its kind in Australia, was recently installed at the Sir Charles Gairdner Hospital (SCGH), Nedlands, WA, with promising early clinical results.^5^

Small‐field dosimetry, known to be challenging due to volume averaging effects and a lack of charged particle equilibrium (CPE), has been extensively discussed in the literature.^6^, ^7^ The problems associated with small‐field dosimetry for flattened beams are likely to be compounded in flattening filter free (FFF) beams, given their inherently higher dose gradients, not just the penumbral region but also in the central beam, and higher doses per pulse.^8^, ^9^

In the context of small‐field SRT, the accuracy of treatment planning systems (TPSs) in predicting dose distributions can be significantly limited by uncertainties in underlying dosimetry data.^2^ In particular, incorrectly measured output factors (OFs) can result in systematic uncertainties leading to incorrect TPS‐derived output.^10^ This would be a major concern when a variable aperture collimator such as the Iris is used, for which its mechanical reproducibility would have to be verified.

Dedicated dosimeters are an essential part of a small‐field‐specific quality assurance (QA) protocol, which has been shown to be clinically justified.^11^ These would ideally have a small water‐equivalent sensitive volume (SV), allowing for high positioning accuracy, and show negligible energy, dose rate, and directional dependence.^12^ Although commercially available detectors do not satisfy all of the above criteria, it has been common practice to perform measurements with at least two types of dosimeters to cross‐check the consistency of results,^13^ as recently recommended by an ICRU report.^6^

For a CyberKnife system, the dosimeter of choice for beam characterization has long been the Gafchromic film, thanks to its small energy dependence and high spatial resolution.^14^, ^15^ Films, though, require a postirradiation analysis process with long waiting times. Film‐derived readings may be affected by large uncertainties due to batch‐to‐batch sensitivity variations, film polarization, nonuniformity, scanning, and handling techniques.^13^

Solid‐state detectors have stable response, a ratio of signal in dosimeter to dose in water that is nearly energy independent in the megavoltage photon range (while calibrated at a depth in water, the same calibration can be used for other depths), high sensitivity and small SVs. Solid‐state detectors thus have the potential to offer comparable performance to Gafchromic film, though with a real‐time read‐out. Their use is recommended by a recent IAEA‐AAPM dosimetry protocol,^7^ but only single detectors used with various scanning techniques have been shown to offer submillimeter spatial resolution.^6^ When used for small‐field dosimetry, correction factors need to be applied to account for beam perturbations, due to their SVs and extracameral components. These factors depend on detector design, treatment head design, beam quality, field size, and measurement conditions.^6^ Monte Carlo (MC) codes are commonly used for modeling linac beam lines, and have been shown to be an effective tool in characterizing detector response in small radiation fields and their required correction factors.^16^ Nevertheless, these remain inconvenient to use in practice, especially for percentage depth dose (PDD), tissue maximum ratio (TMR), and dose profile (DP) measurements because of the multidimensional factor dependencies (field size, depth, and distance).^16^ Most importantly, corrections factors from MC simulations require knowledge of the detector construction and deficiencies in information provided by vendors, or manufacturing variability, will lead to inaccurate results.^17^ A preferable solution would be to design a “correction‐free” detector, or one maintaining a correction factor close to unity. This has been shown to be possible with the addition of low density media to the high density detector components.^18^ However, it would still be necessary to verify that these modifications are appropriate under all beam quality and measurement conditions.^19^

The Octa is a 2nd generation monolithic silicon‐diode array detector designed by the Centre for Medical Radiation Physics (CMRP), University of Wollongong. Its 512 diodes are arranged in four intersecting orthogonal linear arrays such that OF, cross‐plane, in‐plane, and two diagonal DPs are characterized simultaneously with a submillimeter resolution, for any given field size. The Octa was previously characterized as an accurate detector for relative dosimetry under irradiation with both flattened and FFF beams, for small radiation fields as defined with photon jaws.^20^ In the present study, the potential of the Octa for beam characterization in the particular case of small radiation fields for SRT treatments with the CyberKnife system was evaluated.

## 2 MATERIALS AND METHODS

### 2.A The Octa detector

**Figure 2:**
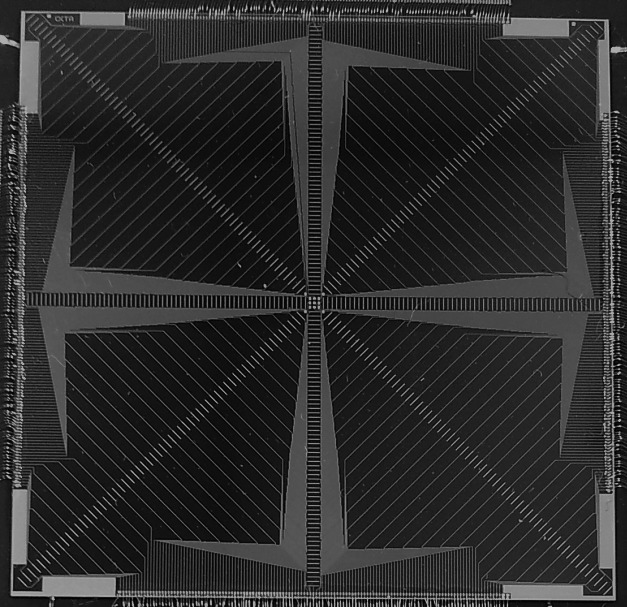
The Octa is a 2D monolithic silicon array detector consisting of 512 diodes operated in passive mode and arranged in four intersecting orthogonal linear arrays. Each diode has a sensitive area of 0.032 mm^2^ with pitch of 0.3 mm along the vertical and horizontal arrays and of 0.43 mm along the two diagonal arrays.

The Octa is a 2D monolithic silicon array detector based on SVs fabricated on a high resistivity p‐type epitaxial layer,^21^ grown on top of a low resistivity p^+^ substrate. A thin protective layer of epoxy covers the SVs. The 512 diodes each have a sensitive area of 0.032 mm^2^. The device (Fig. 2) has a submillimeter resolution with diodes having a 0.3 mm pitch along the vertical and horizontal arrays and 0.43 mm pitch along the two diagonal arrays. The diodes are operated in passive mode and are connected to a multichannel readout electronics data acquisition (DAQ) system based on a commercially available analogue front end (AFE0064, Texas Instruments), which was previously described in detail.^22^, ^23^ An equalization procedure^24^ is used to correct for small differences in each channel response. The Octa is sandwiched between two Perspex plates, each 5 mm thick, with a small air gap on top of its SVs to minimize the number and size of corrections that are required to relate its readings to dose.^25^

### 2.B Experimental measurements

Experimental measurements described in this study were carried out at the Sir Charles Gairdner Hospital (SCGH), Nedlands, WA, Australia, with an Accuray CyberKnife M6 linac. IBA solid water slabs type RW3 were used to reach the required measurement depths.

Measurements by the Octa were compared with those made using a PTW SRS diode 60018 mounted parallel to be them axis in an IBA 3D water‐phantom. The diode was oriented vertically, measuring at the effective point of measurement of 1.3 mm from top surface. Its readings were corrected using the correction factors by Francescon et al.^26^

### 2.C Output factors and dose profiles

In this study, output factors were defined as the ratio between the detector reading at a specific field size (*clin*) and that at the machine specific reference field (*msr*), following the formalism used by Francescon et al.^26^:

$$OF_{\det}=\frac{M^{f_{\mathrm{clin}}}}{M^{f_{\mathrm{msr}}}}$$

where $M^{f_{\mathrm{clin}}}$ and $M^{f_{\mathrm{msr}}}$ are the corrected detector readings in the $f_{\mathrm{clin}}$ and $f_{\mathrm{msr}}$ fields respectively. For the CyberKnife system, the reference field was taken as that given by the 60 mm diameter collimator.

The OFs and DPs were measured by the Octa at 15 mm depth in solid water, 800 mm source to detector distance (SDD). Prior to the measurements, the Octa was aligned with respect to the treatment machine central axis (CAX) by maximizing the response of its central pixel using the smallest available field size (5 mm diameter). Once aligned, for any given field size, OF and DPs (in‐plane, cross‐plane, and two diagonals) were measured simultaneously.

For OF measurements, the detector reading at each field size was taken as the average response of its central pixel over three repetitions of the same measure. This was followed by normalization of these averages to the average reading at the reference field size.

For DP measurements, the Octa reading at each field size was taken as the reading of each channel averaged over three repetitions of the same measure followed by normalization of the response of each channel to the median response of the pixels within 0.5 mm of the CAX. For a quantitative estimation of the FWHM and penumbra width, all profiles were analysed with MATLAB (Mathworks, Inc., Natick, MA, USA) using a shape preserving interpolant function. Penumbra width was taken as the distance between the 80% and the 20% isodose levels.

Following the approach recommended by the vendor,^3^ and as requested by the CyberKnife system TPS, for any given field size DPs were measured at different angles with respect to the in‐plane direction. For the fixed cones, the representative equivalent circular profile was then taken as the average of the profiles measured at 0° and 90°, while for the Iris it was taken as the average of the profiles measured at 0°, 15°, 90°, and 105°, to sample the underlying collimator asymmetry. For both OFs and DPs measurements, circular field sizes investigated were 5, 7.5, 10, 15, 20, 25, 30, 60 mm diameter for the fixed cones and 5, 7.5, 10, 12.5, 15, 20, 25, 30, 60 mm diameter for the Iris. Field sizes were defined at 800 mm from the linac target.

### 2.D Percentage depth dose and tissue maximum ratio

CAX PDDs were measured by the Octa at 800 mm source to surface distance (SSD) with 10 cm solid water for backscattering purposes, reaching the desired water by adding the required amount of solid water slabs on top of the detector. A 60 mm diameter circular field size was investigated for a fixed cone and the Iris. SSD was maintained by moving the linac head.

CAX TMRs were measured by the Octa at 800 mm SDD with 10 cm solid water for backscattering purposes, reaching the desired water by adding the required amount of solid water slabs on top of the detector. 5 and 60 mm diameter circular fields were investigated for fixed cones and the Iris. SDD was maintained by moving the linac head.

Nominal solid water depths were converted to water equivalent depths including accounting for the density of Perspex plates. For a quantitative estimation of the percentage differences, measured values were analysed with MATLAB using a shape preserving interpolant function.

### 2.E Monte Carlo GEANT4 application

Calculations with GEANT4 (GEometry ANd Tracking 4),^27^ a general purpose MC tool‐kit for the simulation of the passage of particles through matter which has been validated for medical applications by different groups,^28^, ^29^ were added to the study to support the experimental characterization of the detector response.

The International Atomic Energy Agency (IAEA) phase space (PHSP) files containing the detailed description (position, direction, kinetic energy, statistical weight, type) of the particles scored at the exit of the Iris collimator, for a CyberKnife linac, were downloaded from the online repository (http://www-nds.iaea.org/phsp/phsp.htmlx). The PHSP files, previously validated by Francescon et al.,^30^ were read by a GEANT4 application purposely developed in‐house for this study using a C++ class adapted from a previous work by Cortés‐Giraldo.^31^ The PHSP files were in this way used as the primary generator in the GEANT4 application in order to simulate the irradiation of a solid water phantom. The solid water was modeled as the IBA type RW3, to match that used for the experimental measurements with the Octa. The GEANT4 Standard EM physics list option 4 was used in this study, with production cuts set to 0.1 mm for electrons and photons in the phantom.

## 3 RESULTS

### 3.A Output factors

**Figure 3:**
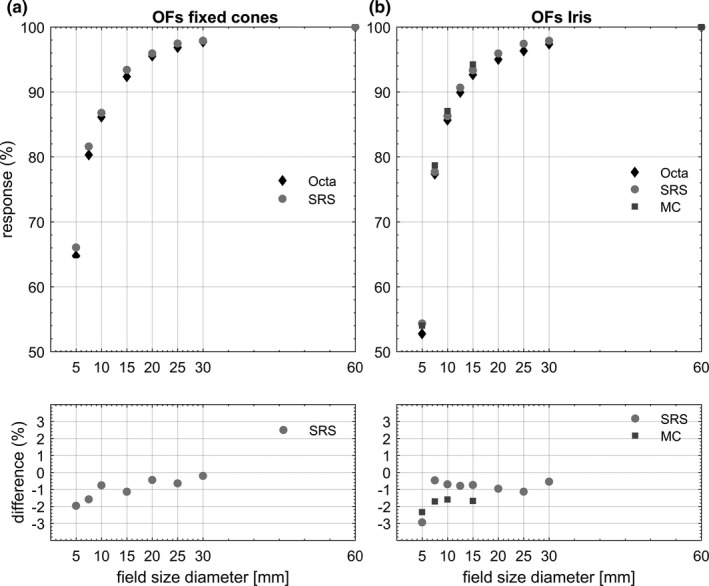
(a) OFs measured by the Octa and SRS diode, with percentage differences with respect to the SRS diode, for fixed cones. (b) OFs measured by the Octa and SRS diode, and MC calculated OFs in solid water, for the Iris. Percentage differences are for the Octa with respect to the SRS diode and for the Octa with respect to MC OFs respectively.

The OFs for the Octa, SRS diode, and MC calculations are shown in Fig. 3, along with percentage differences in the lower panels. MC calculated OFs were taken as the dose deposited in a voxel of solid water whose dimensions were those of the central SV of the Octa detector. When measuring OFs, the central pixels of the Octa were small enough to identify the CAX position accurately without any volume‐averaging effect. Error bars, calculated as 3 standard deviations, did not exceed the symbol size for both experimental measurements and MC calculated results.

### 3.B Dose profiles

**Figure 4:**
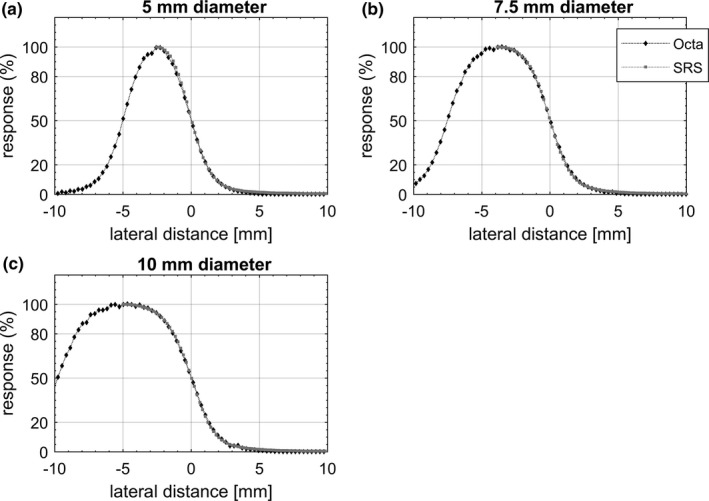
In‐plane and cross‐plane averaged dose profiles measured by the Octa and SRS diode for (a) 5 mm, (b) 7.5 mm, and (c) 10 mm diameter circular field sizes collimated with fixed cones. Profiles are aligned to the 50% response.

**Figure 5:**
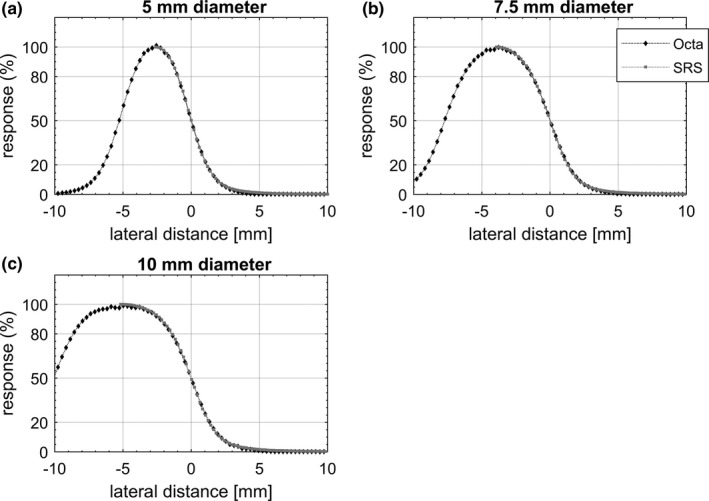
In‐plane, cross‐plane, 15° and 105° degrees averaged dose profiles measured by the Octa and SRS diode for (a) 5 mm, (b) 7.5 mm, and (c) 10 mm diameter circular field sizes collimated with the Iris. Profiles are aligned to the 50% response.

**Figure 6:**
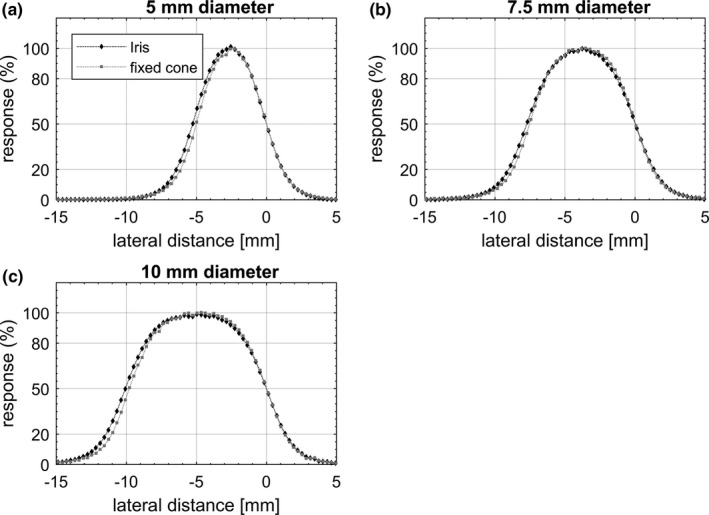
Representative equivalent dose profiles measured by the Octa for (a) 5 mm, (b) 7.5 mm, and (c) 10 mm diameter circular field sizes collimated with fixed cones and the Iris. Profiles are aligned to the 50% response.

**Figure 7:**
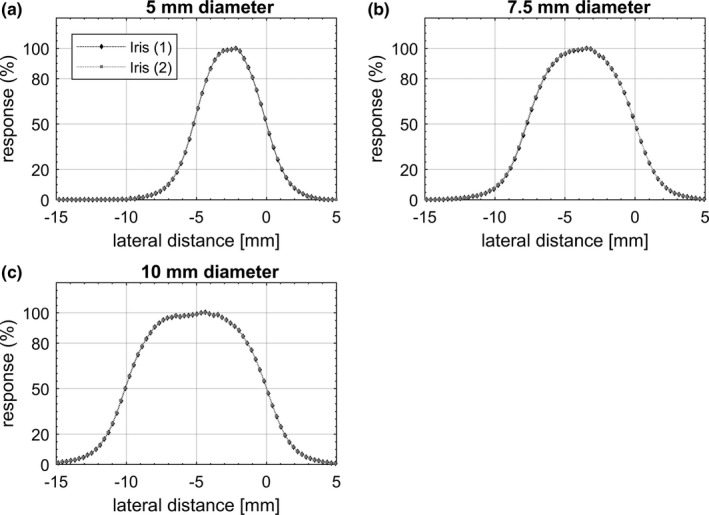
In‐plane dose profiles measured by the Octa before (1) and after (2) a reset of the Iris collimator, for (a) 5 mm, (b) 7.5 mm, and (c) 10 mm diameter circular field sizes. Profiles are aligned to the 50% response. In the DP relative to the 10 mm diameter, a small asymmetry attributed to the non‐perfect uniformity of the detector response could be appreciated.

Representative equivalent circular profiles for the Octa and SRS diode are shown in Fig. 4 for fixed cones and in Fig. 5 for Iris collimated radiation fields. In Fig. 6, equivalent profiles measured by the Octa for fixed cones are compared to those measured for the Iris, for the same nominal field size. In Fig. 7 in‐plane nonaveraged profiles measured by the Octa are compared before and after a reset of the Iris, defined as setting the aperture of the collimator to the desired size, followed by its complete closure and then a reset of the aperture to the desired size.

Profiles are shown in the figures aligned such that the origin lies at the coordinate corresponding to the 50% response. Error bars, calculated as 3 standard deviations, did not exceed the symbol size. FWHM and penumbra values are shown in Table 1 for fixed cones and in Table 2 for the Iris.

**Table 1:** Summary of FWHM and penumbra values measured by the Octa and the SRS diode for radiation fields defined by fixed cones. Values refer to representative equivalent profiles measured at 15 mm depth, 800 mm SDD

Field size diameter (mm)	Octa		SRS diode		Difference	
	FWHM (mm)	Penumbra (mm)	FWHM (mm)	Penumbra (mm)	ΔFWHM (%)	ΔPenumbra (mm)]
5	5.0	2.1	5.0	2.0	0.0	0.1
7.5	7.5	2.4	7.7	2.2	−2.6	0.2
10	9.8	2.6	9.9	2.5	−1.0	0.1

**Table 2:** Summary of FWHM and penumbra values measured by the Octa and the SRS diode for radiation fields defined by the Iris. Values refer to representative equivalent profiles measured at 15 mm depth, 800 mm SDD

Field size diameter (mm)	Octa		SRS diode		Difference	
	FWHM (mm)	Penumbra (mm)	FWHM (mm)	Penumbra (mm)	ΔFWHM (%)	ΔPenumbra (mm)
5	5.2	2.1	5.2	2.1	0.0	0.0
7.5	7.7	2.7	7.8	2.5	−1.3	0.2
10	10.0	2.8	10.3	2.7	−2.9	0.1

### 3.C Percentage depth dose and tissue maximum ratio

**Figure 8:**
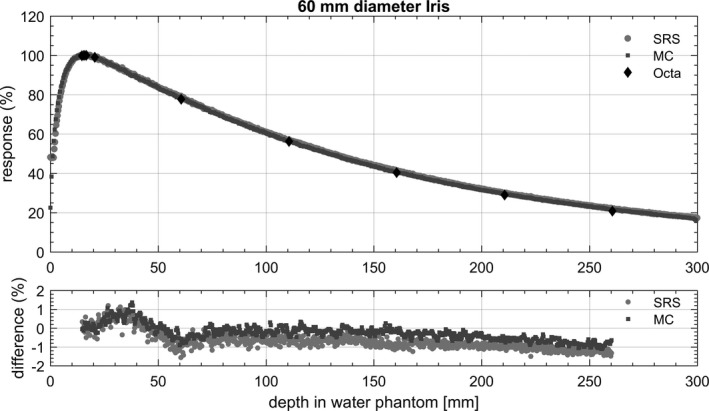
PDDs measured by the SRS diode in water and by the Octa in solid water, along with PDD simulated with MC in solid water (type RW3), for 60 mm diameter Iris. Percentage differences are for the Octa with respect to SRS diode and MC respectively.

**Figure 9:**
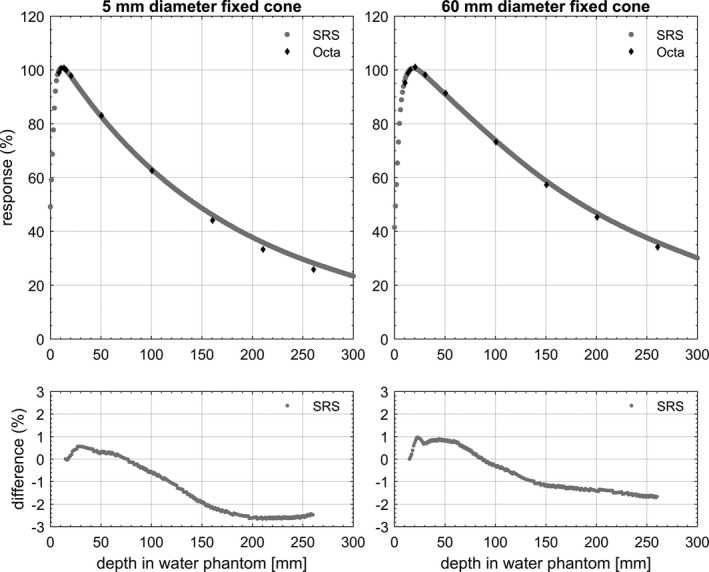
TMRs measured by the Octa in a solid water (type RW3) and SRS diode in water, for 5 and 60 mm diameter fixed cone. Percentage differences are for the Octa with respect to SRS diode.

**Figure 10:**
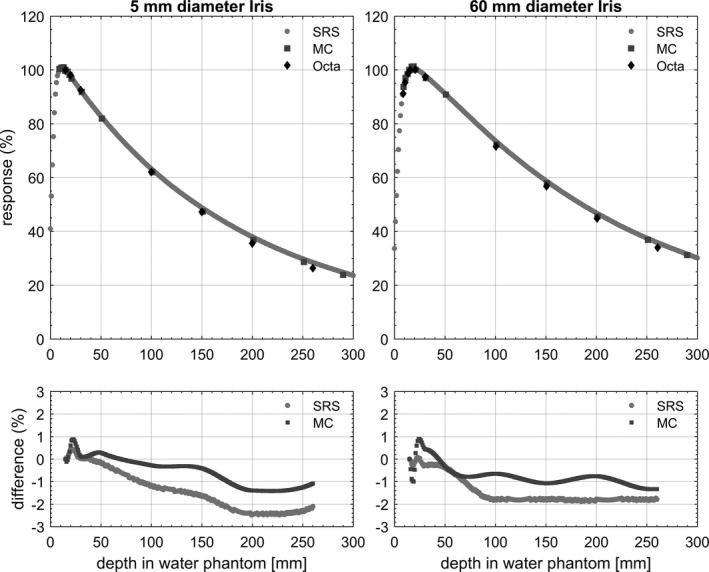
TMRs measured by SRS diode in water and by the Octa in solid water, along with MC simulated values in solid water, for 5 and 60 mm diameter Iris. Percentage differences are for the Octa with respect to SRS diode and MC respectively.

Figure 8 shows the depth doses measured by the Octa in solid water, the SRS diode in water tank and MC calculated in solid water for the 60 mm diameter Iris. Figure 9 shows the TMRs measured by the Octa in solid water and SRS diode in water tank for the 5 mm and the 60 mm diameter fixed cones. Figure 10 shows analogous results for Iris collimated field sizes, with the addition of MC calculated dose depositions. For all results, percentage differences for the Octa with respect to the benchmarks are shown in the lower panels of the corresponding figure.

Error bars, calculated as 3 standard deviations, did not exceed the symbol size for both experimental measurements and MC calculated results.

## 4 DISCUSSION

### 4.A Output factors

Silicon diodes are known to require corrections for output factor measurements due to the electron spectra being perturbed in silicon with respect to water, an effect that increases with decreasing field size. This perturbation has been attributed to the atomic number, mean excitation energy (*I‐value*) and density of silicon SVs being different from that of water, with the nonsilicon extra‐cameral components of the detector playing a non‐negligible role.^32^, ^33^ FFF beams, which have a lower average beam energy than corresponding flattened beams,^9^ may require a different correction factor.

In this study, the Octa OFs were accurate within 3% with respect to the SRS diode for both fixed cones and the Iris, with a maximum discrepancy of 2.9% found for the 5 mm diameter Iris. Discrepancies for the Octa with respect to the expected MC simulated OFs in solid water were well within 2%, except for the 5 mm circular field size for which it was 2.3%.

This conclusion supports the current ‘correction‐free’ design of the detector for the 6XFFF beam quality with a CyberKnife linac. Thanks to the negligible beam quality variations among the different CyberKnife linacs, even of different generations,^34^ we expect this result to extend to all CyberKnife systems currently in operation. Nevertheless, the results show a small but systematic under‐response by the Octa, suggesting that a small adjustment of the air cavity may reduce the discrepancy further.

OFs for the two smallest apertures, 5 and 7.5 mm diameter, were lower for the Iris than for the fixed cones. This result has already been reported in the literature and was attributed to the increased length of the Iris leading to a difference in the head scatter component.^35^ After a reset of the Iris, OFs were accurate within measurement error, an indication of the robust mechanical properties of the collimator. Ideally, this would have to be a long‐term test.

### 4.B Dose profiles

Small irregularities in the profiles measured by the Octa are due the applied equalization procedure not being able to completely correct for the nonuniform sensitivity of the 512 diodes.

Overall, FWHM values for the Octa for in‐plane, cross‐plane, and diagonal DPs were well within 3% with respect to the SRS diode values. In particular, for the fixed cones, a maximum discrepancy of 2.6% in FWHM was found for the 7.5 mm diameter field, with differences in penumbra within 0.2 mm for all fields investigated. For the Iris, a maximum discrepancy of 2.9% in FWHM was found for the 10 mm diameter aperture, with differences in penumbra within 0.2 mm for all apertures investigated.

When comparing equivalent profiles measured by the Octa for fixed cones against those measured for the Iris, all discrepancies were within the spatial resolution of the device of 0.3 mm. In particular, with DPs analysed with MATLAB using a shape preserving interpolant function, a maximum difference of 4% in FWHM was found for the 5 mm aperture (0.2 mm), along with a 2.7% difference for the 7.5 mm aperture (0.2 mm) and 2% difference for the 10 mm aperture (0.2 mm). Penumbra values were accurate within 0.2 mm. These results, which were supported by equivalent SRS diode measurements, were consistent with those of a previous investigation in which FWHM and penumbra values for fixed cones and the Iris were found to be in substantial agreement, with a maximum discrepancy of 0.2 mm in penumbra width for the 5 mm diameter.^36^ By the vendor's technical specifications, the average penumbra for the Iris is expected to be larger by 0.2 to 0.6 mm than that for the equivalent fixed cone and to increase with field size, a consequence of the stepwise approximation of a divergent collimator shape because of the increase in transmission penumbra.^3^ To our knowledge, no other intercomparison between Iris and fixed cones collimator dose profiles exists in the literature.

The Iris collimator is designed to achieve an aperture reproducibility of 0.2 mm at 800 mm SDD,^3^ with the current recommendation (Accuray Physics Essentials Guide 2012, P/N 1023868‐ENG A) for QA suggesting monthly film measurements of all 12 field sizes. Nonequivalent DPs reproducibility after a reset of the Iris aperture were found to be accurate within 2% for all profiles, with a maximum discrepancy of 1.9% for the 5 mm diameter in‐plane profile (<0.1 mm) and of 1% for the 10 mm diameter in plane and cross‐plane profiles. Discrepancies in penumbra values were not appreciable.

### 4.C Percentage depth dose and tissue maximum ratio

For silicon detectors, a decrease in sensitivity is expected with decreased dose per pulse.^37^ To some extent, this effect could be offset by an overestimate of the dose due to the increase of the relative number of low energy scattered photons with increasing depth.^38^, ^39^, ^40^ Although a DPP dependence was found in a previous characterization of the Octa,^20^ in this study discrepancies in PDD with respect to the SRS diode and the calculated MC values in solid water were within 2% at all depths. For these measurements, no corrections were made for dose rate response variations.

By definition, in TMR measurements in the field sizes remain constant with depth and thus the correction factor needed for the Octa remains unchanged related to the change of field dimensions. This is reflected in the TMR plots, where a dose rate dependence becomes apparent leading to a clear under‐response of the Octa beyond 10 cm depth. Nevertheless, TMRs measured by the Octa were in agreement within 3% at all depths with respect to the SRS diode, for both 5 mm and 60 mm circular field diameters with fixed cones. Comparable agreement was found with respect to the SRS diode and MC simulations in solid water for the 5 and 60 mm diameter with the Iris.

### 4.D General observations on the measurements by the Octa

The CyberKnife used for the present study was not equipped with an InCise multileaf collimator. However, based on our results, we believe that the features of the Octa would be well‐suited to QA for this device.

Allowing for the simultaneous acquisition of dose profiles at 0°, 45°, 90°, and 135°, and of those at 15° and 105° upon rotation, the Octa would greatly reduce the measurement time needed to comply with the vendor's QA protocol, potentially allowing for a more robust implementation of the requirements when including DPs along directions not currently considered. In our study, OFs and DPs for all field sizes investigated were measured by the Octa in less than 10 min for the Iris collimator and in approximately 20 min for the fixed cones. PDD measurements were performed in approximately 25 min for both PDD and TMR, for each field size.

### 4.E Commercially available detectors and the Octa

Examples of commercially available detector array recently proposed for machine‐specific CyberKnife QA are the Octavius 1000SRS (PTW, Germany), the SRS‐Profiler (SunNuclear, USA), the Nonius (QUART, Germany), and the ArcCHECK (SunNuclear, USA).

The Octavius 1000SRS is a 2D array of 977 ionization chambers. SVs have a pitch of 2.5 mm in the square central area of 5 cm side, and a 5 mm pitch outside. In a recent characterization of the device,^41^ differences between OFs measurements by the 1000SRS, a synthetic diamond (TM60019, PTW) and a small‐field diode (ETM60017, PTW), were approximately 3.0% for a 5 mm collimator and 1.5% for a 7.5 mm collimator, in agreement with previous investigations.^26^ The size of the SVs (2.3 × 2.3 × 0.5 mm^3^) would be responsible for the 3% under‐response for the 5 mm collimator owing to volume‐averaging effect.^41^ The array sensitivity was investigated by introducing beam shifts by moving the robot with 0.1 mm steps (for the 5, 35, 60 mm diameter fields). The shifts were detected with sub‐mm accuracy.^41^

In a different study, the 1000SRS, the SRS‐Profiler (125 diodes arranged in a star‐like fashion with 4.0 mm resolution) and the Nonius (diodes arranged in a linear array with 2.8 mm resolution), were all able to detect beam shifts with sub‐mm accuracy.^42^ When compared to the other two tested devices, however, the performance of the 1000SRS was found to be superior, comparable to EBT3 films in terms of accuracy and sensitivity, and more user‐friendly.

The ArcCHECK is a 3D cylindrical array of 1386 diodes (0.8 × 0.8 × 0.03 mm^3^) with 10 mm pitch. The EDGE diodes response, a similar version of the ArcCHECK's diodes, was investigated^43^ in CyberKnife small‐fields. OFs agreed with MC calculations and measurements by benchmark detectors within 1% for field sizes larger than 10 mm diameter. Differences were between 3.6% and 5.1% for cones with diameter <10 mm. The ArcCHECK was recently investigated for commissioning of a Multiplan^®^ Monte Carlo dose calculation algorithm.^44^ It was found that while the ArcCHECK addresses some of the small‐field dosimetry challenges (its diodes have real‐time response, high sensitivity and sub‐mm lateral size of the SVs), the measurement of field sizes with diameter inferior or equal to the SVs pitch should be considered with care.

When considering machine‐specific QA applications for the smallest field sizes offered by a CyberKnife (5, 7.5, and 10 mm diameter), the 1000SRS is probably the most obvious choice. The Octa array offers a comparable performance for OFs measurements, without the volume averaging effect of the former, with a superior nominal spatial resolution for DP measurements and most importantly pulse‐per‐pulse real‐time acquisition.

## 5 CONCLUSIONS

In this work, the Octa detector has been investigated for the dosimetry of small radiation fields as used in SRT with the CyberKnife system. For any given field size, the Octa allowed for the simultaneous real‐time read‐out of OFs and dose profiles for cross‐plane, in‐plane, and two diagonal directions. PDD and TMRs were accurate within 3% with respect to both SRS diode and MC simulations, for all field sizes investigated. The Octa was used for a real time high‐spatial resolution verification of the Iris variable aperture reproducibility in terms of FWHM and penumbra values of the dose profiles, as well as OFs. The Iris reproducibility was found to be within the vendor's technical specifications.

Overall, the Octa was shown to be a ‘correction‐free’ dosimeter for routine QA for a CyberKnife system, offering a reliable real‐time read‐out along with unique properties for dosimetry verification, such as a long‐term stability evaluation of the Iris collimator.

## CONFLICTS OF INTEREST

The authors have no relevant conflicts of interest to disclose.
